# Chilblains-Like Lesions in Pediatric Patients: A Review of Their Epidemiology, Etiology, Outcomes, and Treatment

**DOI:** 10.3389/fped.2022.904616

**Published:** 2022-06-23

**Authors:** Jessica Fennell, Karen Onel

**Affiliations:** ^1^Pediatric Rheumatology, Hospital for Special Surgery, New York, NY, United States; ^2^Department of Pediatrics, New York Presbyterian-Weill Cornell, New York, NY, United States

**Keywords:** SARS-CoV-2, chilblain-like lesions, vasculitis, COVID toes, pediatric, review

## Abstract

Vascular pathologies associated with SARS-CoV-2 infection are poorly understood. Color and sensory changes to the extremities, often referred to as “COVID toes” or chilblains-like lesions, have been widely reported in children and adolescents since the onset of the pandemic, raising the concern that they could be a vasculitis secondary to the infection. However, it is unclear if the lesions are a result of the infection or an epiphenomenon. Most literature focuses on adults, and while there are reports on children and adolescents, many of them are small. This review will help medical care providers better understand the epidemiology, etiology, outcomes, and potential treatments for chilblains-like lesions seen in children and adolescents during the pandemic.

## Introduction

The severe acute respiratory syndrome-coronavirus-2 (SARS-CoV-2) first appeared in China in late 2019 and was declared a pandemic within months (1). It has infected over 437 million people and has caused over 5.9 million deaths (2). Following the onset of the pandemic, reports of acral skin changes, often referred to as chilblains-like lesions or COVID toes, emerged in children and adolescents.

Chilblains is an uncommon condition in pediatric patients, and its prevalence and incidence are not well reported (3, 4). Chilblains occurs most notably in people exposed to the cold and damp, as well as those who participate in cold weather sports such as hockey, figure skating, and speed skating (4). In areas that tend to be drier and/or warmer, the condition is rarely reported; only eight pediatric cases were noted in a 10-year retrospective study done at an academic, ambulatory care center in Colorado (5).

Following a cold exposure, chilblains lesions commonly manifest as cold, cyanotic nodules and papules that may evolve to include swelling, pain, pruritis and ulcerations (3). Extremities are commonly affected, particularly fingers and toes, as well as the ears and nose (3).

Chilblains may be the first symptom of an underlying condition, such as anorexia, antiphospholipid syndrome, Sjögren syndrome, rheumatoid arthritis, Behçet syndrome, STING-associated vasculopathy of infantile onset, IRAK4 deficiency, systemic lupus erythematosus, Aicardi-Goutierès or Familial chilblain lupus (3, 6). The latter two are rare conditions caused by mutations in the TREX1 gene and manifest with chilblain lesions following cold exposure (3, 7).

Chilblains also occurs during infection with viruses that have interferon signatures, such as parvovirus B19 and Cytomegalovirus (8, 9). As a high interferon signature in SARS-CoV-2 has been observed, there has been concern that chilblains could be a vasculitic manifestation of SARS-CoV-2 infection (10).

However, few patients with chilblains-like lesions during the pandemic have confirmed cases of SARS-CoV-2. This has generated debate about the etiology and significance of the lesions. This article will review the epidemiology of affected patients, the clinical and pathologic findings of the lesions, the current hypotheses about lesion pathophysiology, patient outcomes, and the implications of these findings.

## Methods

### Search Strategy

Pubmed and Medscape were queried with the terms “pediatric,” “adolescent,” “COVID-19,” “SARS-CoV-2,” “COVID toes,” “vasculitis,” “pernio,” “chilblains,” “pseudochilblains,” and “Raynaud” from database inception to 1/18/22.

### Inclusion Criteria

Inclusion criteria include a pediatric study population, defined as ≤ 18 years old, the ability to separate the SARS-CoV-2 infection data of pediatric from adult cases if reported together, and potential mechanisms of lesion etiology, regardless of patient age.

### Exclusion Criteria

Articles were excluded if the article was not written in English, the study cohort age was >18 years old or not reported, SARS-CoV-2 status of pediatric cases was not separable from adult cases, the primary focus of the paper was not on chilblains-like lesions, or if it was a review.

## Results

**Figure 1:**
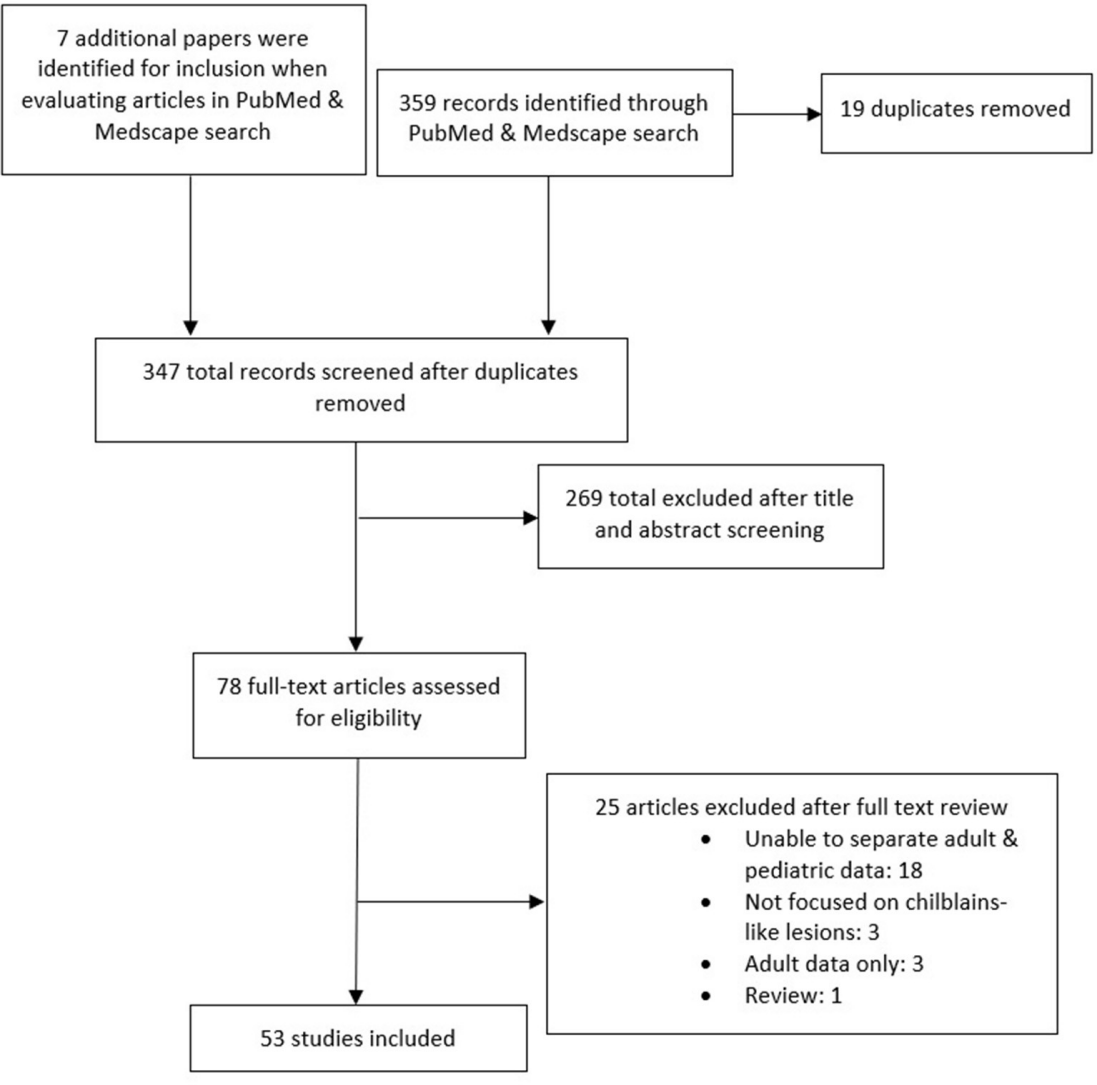
Flowchart of the study selection process.

The literature search generated 359 results. An additional seven articles were found while looking at the articles from the search. Following screening, 53 articles were included in this review. See Figure 1 for compete details. In total, 1,059 reports of chilblain-like lesions were reported (6, 10–57).

### Characteristics of Lesions

Eight-hundred and twenty-one lesions appeared as pink to erythematous to violaceous with a purpuric component (6, 11, 12, 14–17, 19–21, 23, 24, 26–44, 46–48, 51–55). Supplementary Table S1. Three lesions were reported as blue or bluish-red (16, 23, 44). In 470 cases, they were macules, papules or a combination of the two; plaques or patches were also reported in 27 cases (12, 16, 17, 26, 28, 29, 31, 38, 40–43, 46–48, 51, 54, 55). Eight lesions were macules or patches, 6 were reported as nodules, 1 was papulopustular, and 1 was reported as palpable (23, 30, 33, 35, 38). One hundred and fifty-six lesions were reported as chilblain or chilblain-like (14, 18, 25, 33, 48–50, 56, 57). Six were described as dusky (6, 19, 20, 55). Seventeen were described as erythematous, purpuric, or cyanotic (22). Two hundred and forty-five lesions were edematous, and there were five cases of dactylitis (11, 12, 15, 19, 20, 24, 27, 31, 34, 38, 39, 41, 48, 55). Other findings included 68 cases of erosions, 65 cases of vesicles, and 63 cases of blisters/bullae (6, 11, 13, 15–17, 20, 26, 27, 31, 35, 37–39, 42, 43, 47, 48, 51, 54). There were 24 cases with lesion blanching, and 23 cases with ulcers (10, 11, 13, 22, 38). Desquamation was reported in seven cases, and in 20 cases, desquamation, peeling, scabs, and scale were reported together (11, 22–24, 26, 33, 34). Less common findings included crusts (14), acrocyanosis (13), hyperhidrosis (10), livedo or livedo-like (8), acrorhigosis (7), pulpitis (7), eccrine hidradenitis-like (6), telangiectasia (4), acrocholosis (3), urticaria (3), pustules (3), erythema nodosa (2), targetoid (2), cheilitis (1), cyanotic lesions (1), scale (1), and petechiae (1, 10, 17, 19–21, 26, 28, 40, 43, 47, 48, 51, 54). In 65 cases, the rash morphology was unspecified (11). Of note, many of these features were also reported in unspecified numbers: see Supplementary Table S1 for more detail.

Lesions were located on the feet in 728 cases, simultaneously on hands and feet in 109 cases, and hands only in 101 cases (6, 10–55, 57). There was a predilection for the toes and heels: 154 and 40 cases respectively (6, 10–13, 16–18, 20–23, 26, 27, 29–33, 35–38, 40–42, 46, 51, 53–55). Additionally, 34 lesions were reported on the soles, 26 on the dorsum of the feet, 12 on the fingers, nine on the palms, and seven on the dorsum of the toes (6, 12, 16, 19–22, 26, 28–31, 33–38, 40, 42–44, 51, 53, 55). Other locations included the dorsum of the hands in three cases, the ankles in three cases, the face in two cases, and the lateral aspects of the feet in one case (6, 12, 16, 18–21, 23, 26, 33, 37, 38, 42, 44, 54, 55). Lesions were infrequently reported on the axillae, fingertips, thighs, trunk, and tips of toes: all of these locations had one occurrence reported (26, 28, 33, 34, 43). There was an unspecified number of lesions located on the hypothenar, thenar, and nailfold area, while the lesion location was not specified in 35 cases (11, 20, 34, 38, 47, 56). Of note, there were reports of lesions without specified numbers; see Supplementary Table S1 for more details.

One study reported that the majority of lesions were on feet (96.3%), followed by hand lesions (11.9%), and lesions on the head or the neck (11.4%) during the spring of 2020 (11). Among patients with feet lesions, toes were the most commonly affected area, but changes were also noted on the periungual areas, heels, and the dorsum of the feet (11).

Though some lesions were asymptomatic, there were reports of pain in 334 cases and pruritis in 335 cases both prior to and with the onset of the lesions (6, 11–15, 17–20, 23–25, 27–30, 32–38, 40, 42, 44, 46, 48, 50, 51, 53–55, 57). In nine cases, pain, pruritis, and swelling were reported together (22). Temperature change was reported in 42 cases, tingling in 24 cases, burning in 22 cases, and allodynia in 10 cases (11, 19, 22, 24, 32, 34, 35, 38, 44, 51, 54). In 24 cases, lesions were reported as non-pruriginous (13). Unspecified symptoms of rash were reported in 17 cases (11). Many articles reported unspecified counts of these symptoms: see Supplementary Table S1.

On dermoscopy, there were 12 reports the background color of the lesions as pink to erythematous to violaceous erythema; a coppery red was also described in six cases (6, 34, 39). Additional findings included rosettes in 31 cases, red dots in 29 cases, dotted vessels in 26 cases, white streaks in 25 cases, capillary dilation in 15 cases, abnormal capillary morphology in 10 cases, pigmented areas in nine cases, vessel ectasia in nine cases, scales in eight cases, hyperpigmentation in seven cases, microhemorrhages in seven cases, unstructured areas with color changes in seven cases, and hemorrhagic dots in six cases, edema in five cases (6, 22, 39, 51, 54). There were winding vessels in three cases, irregular linear vessels in two cases, cuticular vessels in two cases, linear vessels two cases, crusts intwo cases, and desquamation in two cases (22, 39, 51). There was one case of each of the following: vessels with branches, reduced capillary density, bullae, and multiple short vessels in a perpendicular arrangement (39, 51). An unspecified number of ischemic areas and purpuric dots were reported (6) Supplementary Table S1.

The gross descriptions of biopsies noted edema in 33 cases (6, 10–12, 18, 29, 31, 33, 35, 41). Additional findings included vacuolar changes in 36 cases, spongiosis changes in 27 cases, mucin deposits in 22 cases, red cell extravasation in 18 cases, fibrin deposition in eight cases, purpura in seven cases, vascular ectasia in three cases, dilated blood vessels in two cases, necrosis in two cases, and degeneration of endothelial cells in one case (6, 10, 11, 18–20, 22, 26, 35, 41, 45, 51, 52). Eight biopsies demonstrated swollen endothelial cells, and an unspecified number of samples had tubule-reticular inclusions within the endothelial cells (10, 18, 19, 41). Twenty-two thrombi, of which 3 were fibrin thrombi, and four were microthrombi, have also been identified (6, 18, 19, 26, 41, 45, 51, 52). Lymphocytic and histiocytic infiltration of vessel walls was also reported in 12 cases (10, 17, 18, 20). Some reports grouped findings into categories: there were 12 reports of lymphocytic vasculitis, two reports of thrombogenic vasculitis, and 4 reports of chilblains-like lesions (11, 17, 18, 26, 41, 51, 52) Supplementary Table S2.

Lymphocytes were commonly reported as the dominant tissue infiltrate and were present in 129 instances; CD3+ T lymphocytes were reported in 14 samples (6, 10–12, 17–20, 22, 29, 31, 33, 35, 41, 45, 51, 52). The infiltrate was perivascular in 75 cases, perieccrine in 42 cases, and periadenexal in 3 cases (6, 10–12, 18–20, 22, 31, 33, 35, 41, 45, 51, 52). CD20+ B cells were also reported in 12 instances (18). One study identified an increased expression of IFN-α, MCP-1, and CXCL10 (47). Supplementary Table S2.

Deposition of complement was present in 26 cases, and there were 5 reports of immunoglobulin M, and 1 report of immunoglobulin A (11, 26, 45, 52). Two studies reported the presence of viral structures that could be SARS-CoV-2 particles (18, 26). However, biopsies were negative for SARS-CoV-2 in 44 samples (22, 33, 43, 47, 51, 52). One study reported a positive SARS-CoV-2 testing on a chilblains-like lesion, and there were an additional eight positive tests on other biopsies (18, 33, 45). Additional details about the histopathology are detailed in Supplementary Table S2.

### Characteristics of Patients

Chilblains-like lesions have been reported in children younger than 1 year old, though most cases were in late childhood to early adolescence (11). Table 1. In a Spanish cohort including 22 patients, Andina et al. found that a majority of children affected with chilblains-like lesions were between the ages of 10 and 13 years old; 14 to 17 years old children were the second most common group, and patients 9 years old and younger were the least frequently affected group (6). Caselli et al. reported an Italian cohort with 38 cases (16). Ages ranged from 7 to 18 years old, with a median age of 13.5 years old (16). In a multi-country pediatric dermatology registry, 378 children had a mean age of 13 years old (11).

**Table 1:** Patient demographics.

**Age**	
Average mean (years)^*^	12.11
Average median (years)^*^	13.18
Range (years)	0–18
Sex	
Female	402
Male	575
Unknown/not reported	82
Race	
American Indian or Alaska Native	2
Asian	43
Black or African American	7
More than one	11
Native Hawaiian or other Pacific Islander	0
White/Caucasian	332
Unknown/not reported	664
Ethnicity	
Hispanic or Latino	19
Non-Hispanic or Latino	277
Unknown/not reported	763
Nationality	
American	269
Argentinian	2
Australian	4
Belgian	14
British	21
Canadian	89
French	130
Irish	2
Italian	362
Kuwaiti	1
Moroccan	1
Spanish	156
Welsh	1
Unknown/not reported	7
Total number of patients	1,059

* *Average median age was calculated when data was available, and in studies in which only the mean was reported, that data is represented by the average mean age*.

Most studies have reported a male predominance. Castelo-Soccio et al. reported 229 males and 148 females affected by chilblains-like lesions (11). Among the case reports and case series reviewed, there were 326 males (57.39%) and 242 females (42.60%) (6, 10–57). Most case reports and series did not report race or ethnicity. Most studies did not specify nationality, but as a majority of articles were case reports or series, the population was assumed to be native to the country unless otherwise indicated. Though not common, there are several reports of siblings having chilblains (6, 20, 23, 24, 41, 49, 51, 54). See Table 1 for additional demographic details.

Reports of chilblains-like lesions began to appear in the literature at the onset of the SARS-CoV-2 pandemic and were suspected to be a result of the viral infection. However, of the patients reported, the majority were negative for SARS-CoV-2: in sum, 499 patients tested negative (47.12%), 64 patients had positive tests (6.04%), and 496 patients were not tested or the data was unavailable (46.84%) as noted in Table 2 (6, 10–57).

**Table 2:** SARS-CoV-2 infection status by serology or PCR testing.

**Study**	**Positive test**	**Negative test**	**Not tested or unknown status**
Castelo-Soccio, et al. (11)^*^	6	134	238
Adina, et al. (6)	1	18	3
Colonna, et al. (12)	0	6	24
Denina, et al. (13)	4	20	0
Fertitta, et al.^§^ (14)	1	15	1
Piccolo, et al. (15)	2	13	48
Caselli, et al. (16)	0	38	0
Brancaccio, et al. (17)	1	1	0
Colmenero, et al. (18)	0	6	1
Colonna, et al. (19)	0	3	0
Cordoro, et al. (20)	0	6	0
Diociaiuti, et al. (21) ^†^	10	3	0
Discepolo, et al. (22)	0	17	0
Feder, (23).	1	2	0
Gallizzi, et al. (24)	0	9	0
Garcia-Lara, et al. (25)^§^	0	11	16
Garrido Ruiz, et al. (26)	0	7	0
Kerber, et al. (27)	1	0	0
Klimach, et al. (28)	1	0	0
Ladha and Dupuis (29)	1	0	0
Landa, et al. (30)	1	0	1
Locatelli, et al. (31)	1	0	0
Mohan and Lind (32)	0	1	0
Neri, et al. (33)	1	0	0
Neri, et al. (34)	0	5	0
Neri, et al. (35)	0	8	0
Nirenberg and Herrera, (36)	1	0	0
Rodríguez-Pastor, et al. (37)^§^	4	17	13
Papa, et al. (38)	11	0	0
Piccolo, et al. (39)^§^	0	3	6
Rafai, et al. (40)	0	1	0
Roca-Ginés, et al. (41)	0	20	0
Rosés-Gibert, et al. (42)	0	7	29
Rouanet, et al. (43)	0	3	0
Ruggiero, et al. (44)	0	0	33
Tammaro, et al. (45)	0	0	1
Tosti, et al. (46)	0	0	2
Vastarella, et al. (47)	0	15	0
Hubiche, et al. (48)^§^	2	30	71
Rizzoli, et al. (49)	1	10	0
Recalcati, et al. (50)	2	0	0
El Hachem, et al. (51)	6	13	0
Herman, et al. (52)	0	14	0
Kluckow, et al. (53)	0	2	2
Fabbrocini, et al. (54)	0	15	0
Colonna, et al. (55)^¶^	1	4	0
Magro, et al. (56)	0	1	0
Jacquin-Porretaz, et al. (57)^‡^	1	5	1
Recalcati, et al. (58)	3	16	6

* *13 patients defined as positive in this study were not tested; they are included in this paper as not tested or unknown*.

§ *Serology and PCR testing were done; each result was counted as a different patient, up to the total number of patients in study, as unclear which patients had the different tests*.

† *Seventeen patients were excluded from these results as they were reported in a prior study (22).*

¶ * Study included 4 previously reported patients, of which 1 developed positive serology testing in this study; that patient is reported here and omitted from original study (19)*.

‡ *Two patients in this study were reported as having photosensitive rashes; they are not included in this paper*.

In most cases, patients did not report systemic symptoms of illness prior to the onset of chilblains-like lesions (11, 14, 17, 20–24, 26, 29, 30, 32, 36, 37, 52, 53, 57). However, there have been reports of systemic symptoms occurring prior to, concurrent with, or following the appearance of chilblains-like lesions. The most common symptoms were fever (116), cough (80), diarrhea/gastrointestinal symptoms (54), and sore throat (6, 11–16, 18–20, 22, 24–28, 30–34, 36–38, 41–44, 48, 50–53, 55, 57) Supplementary Table S3. Additional symptoms included rhinorrhea in 34 cases, malaise in 18 cases, myalgias in 16 cases, COVID-like symptoms in 14 cases, headache in 12 cases, unspecified respiratory symptoms in 11 cases, cough or rhinorrhea in 11 cases, influenza-like symptoms in 10 cases, rhinitis in nine cases, ENT symptoms in seven cases, asthenia in eight cases, conjunctivitis in 8, dyspnea in 5 cases, emesis in 5 cases, anosmia in four cases, upper respiratory tract infection in 4 cases, congestion in 4 cases, coryza in three cases, nausea in 3 cases, pain in 3 cases, ageusia in 2 cases, dysgeusia in 2 cases, chest pain in two cases, and maculopapular rash in 2 cases (6, 11–15, 17–22, 24, 28, 30–34, 36–38, 40, 42, 43, 47, 50–52, 54, 57). There was 1 report of each of the following: arthralgia, back pain, irritable, pharyngitis, abdominal rash, chest rash, face rash, ulcers, chills, cold, erythema multiforme, and fatigue (11, 24, 27, 32, 36, 43, 44, 57). One patient was asymptomatic, but was found to have bilateral pneumonia on x-ray (30) Supplementary Table S3.

Two hundred and ten cases had possible contact with SARS-CoV-2, 116 cases had contact with a known SARS-CoV-2 case, and an additional 13 cases had possible or definite contact with a SARS-CoV-2 contact (10–25, 28, 30, 31, 33, 36, 37, 42, 47–49, 51, 52, 54) Supplementary Table S3.

The illnesses in potential SARS-CoV-2 contacts varied. In one case, the patient's family was sick: one parent had a cough and the other had a flu-like illness (28). Hubiche et al. reported symptoms of illness in 77 families (48). In several cases, patients were exposed to people with upper respiratory tract infection symptoms (12, 20). Three cases had recently returned from international travel (20). One study noted that family members had been ill (headache, gastrointestinal symptoms, cough, or fever) in the 1 to 2 months preceding lesion onset (51).

The majority of cases did not have evidence for other infections, such as *Mycoplasma pneumoniae*, enterovirus, human metapneumovirus, coxsackie virus, herpes simplex virus, Epstein-Barr virus (EBV), Cytomegalovirus (CMV), parvovirus B19, *Bartonella henselae*, and *Rickettsia conorii* (16, 19, 23, 27, 28, 34–37, 47, 49–51). Supplementary Table S4. In the few cases with positive testing, EBV (2 cases with IgM, and 3 cases with IgM and IgG), *Mycoplasma pneumoniae* (2 cases), and *Chlamydia pneumonia* (1) are reported (15, 16, 34, 37, 54) Supplementary Table S4.

The time from systemic symptoms of SARS-CoV-2 development to lesion onset was variable. In 58 cases, systemic symptoms preceded the onset of lesions by at least a week, and in 4 cases, the lesions occurred within 7 days of systemic symptom onset (6, 15–17, 19–22, 24, 27, 30–32, 36, 46, 50, 51, 54, 57). There were also concurrent appearances of lesions and systemic symptoms (7). One study noted 3 cases that had simultaneous or overlapping appearance of lesions and systemic symptoms, while another noted 4 cases in which the time from systemic symptom onset to lesion onset ranged from 0 days to 7 days (6, 42). One study reported systemic symptom onset a mean of 22 days before the lesion onset in six cases, with a range of 5 to 46 days. Another study reported a mean of 12.62 days from systemic system onset to lesion onset (14). Lesions preceded systemic symptoms in 4 cases (14, 26, 51) Supplementary Table S5. Among siblings with a history of chilblains-like lesions, siblings often developed lesions within several days to a week of the index case (20, 23).

A variety of co-morbid medical conditions have been reported, including attention-deficit/hyperactivity disorder in eight cases, asthma in four cases, atopic dermatitis in two cases, and celiac disease in two cases (6, 14, 16, 18, 30, 53). There was one case of each of the following: headaches, allergic rhinoconjunctivitis, seasonal allergies, urticaria, diabetes mellitus, Crohn disease, nephrotic syndrome, alopecia areata universalis, Kawasaki disease, Wolff-Parkinson-White, peripheral neuropathy, drug allergy, and an undisclosed X-linked condition (14–16, 28, 32, 46, 52). Two studies noted a family or personal history of coagulation disorders, though anther study did not find an association (15, 16, 20) Supplementary Table S4.

Raynaud phenomenon and/or chilblains were reported in 12 cases, but several studies did not elicit this history (6, 13, 14, 18, 22, 24, 25, 31, 38, 41, 43, 46, 49, 52, 53). Two studies reported ten cases with autoimmune disease, but specific conditions were not reported (15, 16). One study reported a patient with systemic lupus erythematosus, and another study reported a case with IgA vasculitis (22, 41). One study reported only 18.3% of patients had comorbidities, though the comorbidities were not detailed, with the exception of one patient who was found to have Sjögren disease (11) Supplementary Table S4.

Among studies reporting medication use, 31 patients either did not take medications or had been on stable doses for at least a month before the appearance of chilblains-like lesions (12, 13, 32). One patient had received acetaminophen intermittently around the time of lesion onset, and one patient had recently started ferric sulfate (41). Seven patients with ADHD reported no changes to their treatment in the 6 months prior to lesion onset (6, 13) Supplementary Table S4.

There is little detail reported about family history. There were 5 cases of autoimmune thyroiditis,4 cases of atopy, 3 cases of chilblains, 2 cases of hemolytic anemia during pregnancy, and one report of each of the following conditions: thromboangiitis obliterans, inflammatory bowel disease, dyshidrosis, and hyperhidrosis (22, 34, 35). Several articles reported no autoimmune or inflammatory conditions (11, 20, 24, 35, 54). There were 8 reports of coagulation disorders in cases or in their families (15, 16) Supplementary Table S6.

Twenty-three siblings had lesions: two pairs of brothers (four cases), a pair of sisters (two cases), four pairs of siblings with unspecified sex (eight cases), and three sets of three siblings (nine cases) (6, 20, 23, 24, 49, 51, 54). There were an additional six cases who had unspecified family members with lesions (41) Supplementary Table S6.

While the data was not robust about the lifestyles and activity levels in patients during the SARS-CoV-2 pandemic, 13 cases noted a decrease and 1 case reported a stable amount of activity (52). Fourteen cases were watching more television during the SARS-CoV-2 pandemic than prior to the pandemic (52). One study reported 8 patients going barefoot or only wearing socks during the pandemic (35). Additionally, there were 26 cases of patients walking barefoot and 5 cases of patients walking with socks (41, 51). Routine shoe wear was reported in 1 case; 15 cases reported patients not wearing shoes regularly at home (41, 52). Seven cases reported exposure to cold floors, and 19 cases reported no exposure to cold (35, 53, 54). Accessibility to heating was variable: 10 cases reported living in heated homes, 18 cases reported heating was not available in the home, and 5 cases reported that heating was off (35, 41, 43) Supplementary Table S5.

In a majority of cases, the chilblains-like lesions were asymptomatic or caused mild symptoms (15, 25, 42, 44, 51). For patients with very painful, pruritic, or swollen lesions, treatments included topical steroids in 162 cases, oral analgesics in 49 cases, oral antihistamines in an unspecified number of cases, oral steroids in four cases, and emollients in two cases (1, 6, 11, 13, 23, 29, 32, 35, 36, 38, 44, 46). There were two cases in which a patient was treated with a calcium channel blocker (one topical and one oral); one of these patients also received a topical anticoagulant (11, 24, 44). Additional treatments included topical steroids or antibiotics in six cases, prophylactic heparin in one case, mometasone furoate cream in one case, hydroxychloroquine in 1 case, azithromycin in 1 case, and instructions to avoid cold and/or damp environments in six cases (29, 30, 35, 42, 44). Warming measures were recommended in 74 cases (11). Fifty-seven unspecified treatments were also reported, and 59 cases did not receive treatment (11, 19, 24, 25, 51, 57). A number of treatments were also reported without specified numbers: Supplementary Table S2.

Four cases that had concomitant erythema multiform also received topical and oral steroids (6). Outpatient therapy was usually sufficient for treatment. In one study, a patient with chilblains-like lesions who was not included in the analysis (reported after initial cohort was selected) required hospitalization for treatment of SARS-CoV-2 infection (11).

Some patients tried medications they had at home before visiting a doctor. Treatments included disinfectants in four cases, topical antibiotics and/or corticosteroids in 4 cases, systemic antibiotics or corticosteroids in three cases, and anti-fungal treatments in three cases (22) Supplementary Table S2.

The duration of lesions varied from several days to 145 days, though the majority of cases resolved within several weeks (11–15, 17–19, 22, 23, 25, 26, 28, 29, 33–37, 43, 44, 48, 53) Table 3. In four cases, resolution occurred by day 6, in 47 cases it occurred between 7 to 20 days, and in 80 cases, it occurred after 21 days (11, 13, 17, 19, 22, 23, 25, 26, 28, 29, 33–36, 43, 44, 48, 53). A large study of 378 cases reported lesions resolving an average of 21.6 days after onset, though the median time to resolution was 14 days (11). Other reported outcomes included the following: a majority of 71 cases had a median resolution time of 47 days, 19 cases resolved between 14 and 30 days, 17 cases resolved after a mean of 27 days, eight cases had resolution by a median of 7 days, and seven cases resolved by 8 weeks (12, 14, 18, 37, 48). Unspecified times to resolution are also reported: Table 3.

**Table 3:** Lesion outcomes.

**Study**	**Duration of lesions**	**Lesion status if not healed**
Castelo-Soccio, et al. (11)	14 days (median) & 21.6 days (average): 378	
Andina, et al. (6)		3 to 5 weeks improved or almost complete resolution: 22; 3 to 5 weeks no worsening of lesions: 22
Colonna, et al. (12)	7 days (median) with a range of 1 to 24 days: 8	9 to 39 days lesions ongoing: 16
Denina, et al. (13)	8% lasted < 1 week	ongoing > 14 days: UN
Fertitta et al. (14)	27 days (mean) with a range of 10 to 50 days: 17	15 days: 1 relapse 45 days: 1 relapse
Piccolo, et al. (15)*v*	6.3% resolved quickly: UN	Relapsed: UN; Stable: UN
Brancaccio, et al. (17)	14 days: 1 (first instance)	Relapse: 1
Colmenero, et al. (18)	<8 weeks: 7	
Colonna, et al. (19)	3 days: 2; 5 days: 1	
Discepolo et al. (22)	49 to 145 days: 14; Unspecified time to resolution: 3	
Feder, (23)	6 weeks: 1	
Garcia-Lara, et al. (25)	14.6 days (not specified if mean, median, or mode): 27	
Garrido Ruiz, et al. (26)	2 to 4 weeks: 7	
Klimach, et al. (28)	10 to 14 days: 1	
Ladha and Dupuis, (29)	3 weeks: 1	
Mohan and Lind, (32)		2 months improved: 1
Neri, et al. (33)	2 weeks: 1; 3 weeks: 1	
Neri, et al. (34)	3 to 4 weeks: 5	
Neri, et al. (35)	4 to 5 weeks: 8	
Nirenberg and Herrera, (36)	~1 month: 1	
Rodríguez-Pastor, et al. (37)	16.5 days (unclear if mean or median) with a range of 14 to 30: 19	4 weeks improved: 15
Papa, et al. (38)	12 to 15 days after seen at pain center: 11	
Rouanet, et al. (43)	27 days: 2; 43 days: 1	
Ruggiero, et al. (44)	4 to 16 days: 17	4 days improved: 1
Hubiche, et al. (48)	1 month: 36; 47 days (median): UN	1 month improved: 25; 1 month stable: 8; 1 month worse: 2
El Hachem, et al. (51)		14 days improved: 19
Kluckow, et al. (53)	3 days: 1; 3 weeks: 2; 3.5 weeks: 1	

*UN: unspecified number*.

While lesion improvement followed by resolution was frequently reported, there were reports of outliers. There were 83 cases with improvement, 24 cases with stable or ongoing lesions, three cases with relapse of lesions, and two cases in which the patients worsened (6, 12–14, 17, 32, 37, 44, 48, 51) Table 3. In the study with worsening patients, the rest of the cohort fared well, with stable lesions in eight cases, improvement of lesions in 25 patients, and resolution of lesions in 36 patients (48).

Hyperpigmentation was seen in some lesions during follow up (6, 22, 54). Skin desquamation was rarely reported with lesion healing (22). Nail changes were also noted in some patients. Onychomadesis was observed in two patients 8 to 10 weeks after the onset of skin lesions and was limited to toenails (21) Supplementary Table S1.

## Discussion

This review highlights the lack of SARS-CoV-2 positivity among patients with chilblains-like lesions despite temporal association and the positive outcomes among patients with lesions.

While SARS-CoV-2 testing was limited at the start of the pandemic, the majority of studies that utilized testing later on failed to identify the infection. A possible explanation for this could be that the lesions are secondary to lifestyles changes, leading to alterations in extremity blood flow and in color as seen in other conditions, such a frostbite and trench foot (58, 59). Blood flow may decrease as much as 90% during cold exposure (58). However, some literature suggests that SARS-CoV-2 may be the inciting agent. The spike (S) protein of the SARS-CoV-2 virus binds with angiotensin-converting enzyme 2 (ACE-2) receptor to enter cells, the envelope protein of the virus was shown to amplify inflammasome activation, and the nucleocapsid protein has an immunomodulatory function that could also increase inflammasome activation, possibly further facilitating this process (1, 60). Other studies suggest that SARS-CoV-2 virus may disrupt the communication between endothelial cells and pericytes leading to endothelial cell injury (61).

An increase in interferon is observed in some patients with chilblains-like lesions, though as noted previously, many patients test negative for SARS-CoV-2. Genetic studies done by Frumholtz et al. showed an upregulation in genes associated with natural killer and cytotoxic T cells, both of which occur in the presence of increased interferon (62). The work of Magro et al. also supports the importance of type I interferon signaling in biopsies of chilblains-like lesions in patients, though in this cohort, 2 of the patients had negative testing for SARS-CoV-2 and the third patient was not tested (10). Of note, children have a strong type I interferon response during infection with SARS-CoV-2, and some authors suggest that this response may blunt or entirely suppress the immune system's reaction to the virus (10, 20, 23). There is a lack of detectable infection or symptoms of illness, these children could still develop chilblains-like lesions in response to the high levels of interferon (24, 39).

Despite this possibility, the increased reports of chilblains-like lesions in countries with very low SARS-CoV-2 infection rates raise the possibility of another factor, and possibly multiple other factors, giving rise to chilblains-like lesions (63). In Nordic countries where strict lockdown measures were not initially imposed, there were few cases of chilblains-like lesions (63). When the lesions did occur, patients responded well to topical steroid treatments and lifestyle modifications focused on warming the extremities (63).

Contrary to this, when lockdown measures were in place, an increase in chilblains-like lesions appeared. Four Australian children had chilblains-like lesions during a strict lockdown period (53). At the time, there were 7000 cases of COVID-19 in the country and <1,700 in the region, making an exposure to virus unlikely (53).

An increased number of chilblains-like lesions were also observed in the United States during the first wave of SARS-CoV-2 lockdown (11). Children reported not wearing socks or shoes while doing their schoolwork for prolonged periods of time, which suggests that environmental factors could play a role in the appearance of chilblains-like lesions (11). Additional studies confirmed that many pediatric patients with chilblains-like lesions were not wearing shoes, and only some were wearing socks during the pandemic (35, 41, 51). In a Spanish study, fifteen of twenty patients reported walking barefoot in their homes, and none of their homes were heated during that time (41).

Furthermore, pediatric patients and their caretakers spent more time together during the pandemic, affording caretakers an increased opportunity to notice the lesions. Media reports on the chilblains-like lesions may have increased awareness of these lesions and may have prompted families to seek medical attention (22).

The SARS-CoV-2 pandemic caused, and continues to cause, a great deal of anxiety and stress, both of which may contribute to the development of amplified musculoskeletal pain syndrome (AMPS) (64, 65). This condition may cause changes in skin color and temperature; allodynia may also develop (66). While AMPS does not explain a majority of the reported chilblains-like lesions, it could explain some of the lesions that last for prolonged periods of time and that have an intense sensory component.

It is important to educate families that chilblains-like lesions are not associated with the severe morbidity and mortality that occur with SARS-CoV-2 infection and will resolve without treatment. However, it is equally important that they know the lesions may cause some discomfort and are instructed to seek care for persistent symptoms. The recommendation of supportive care measures, such as warm shoes and socks, are also appropriate as this may prevent the lesions from worsening and may facilitate resolution. If there is concern for AMPS, a trial of physical therapy may be beneficial as increased physical activity is the mainstay of treatment for this condition. Additionally, mental health services may be offered to help ease the psychological burden of the pandemic. If an underlying condition, such as systemic lupus erythematosus, is suspected, a referral to a specialist would be beneficial.

The lack of positive SARS-CoV-2 testing despite the presence of chilblains-like lesions suggests that SARS-CoV-2 infection is not the underlying etiology. The lesions may be incidental findings, possibly secondary to lifestyle and environmental changes. If chilblains-like lesions truly are an epiphenomenon, they may disappear as people resume their activities prior to the SARS-CoV-2 pandemic. Future studies are needed to evaluate the evolution of these lesions. In conclusion, this study supports current literature that the chilblains-like lesions seen during the SARS-CoV-2 pandemic are likely secondary to a multifactorial epiphenomenon as most patients test negative for the virus and that the majority of outcomes are good.

## Author Contributions

JF and KO developed the paper topic and wrote the manuscript. JF did the literature search and literature review. All authors contributed to the article and approved the submitted version.

## Conflict of Interest

The authors declare that the research was conducted in the absence of any commercial or financial relationships that could be construed as a potential conflict of interest.

## Publisher's Note

All claims expressed in this article are solely those of the authors and do not necessarily represent those of their affiliated organizations, or those of the publisher, the editors and the reviewers. Any product that may be evaluated in this article, or claim that may be made by its manufacturer, is not guaranteed or endorsed by the publisher.
