# Supplementary material for: Chilblains-Like Lesions in Pediatric Patients: A Review of Their Epidemiology, Etiology, Outcomes, and Treatment
Source: Front Pediatr. 2022 Jun 23;10:904616. doi: 10.3389/fped.2022.904616 (PMC9259963; doi:10.3389/fped.2022.904616)
Supplement: Supplementary file 1 [file Table_1.DOCX]

Table S1. Dermoscopy, Lesion Features, Location of Lesions, & Sensory Alterations*

| Study | Dermoscopy | Lesions features | Location of lesions^ | Sensory alteration |
| --- | --- | --- | --- | --- |
| Castelo-Soccio L, Lara-Corrales I, *et al.* |  | Bullae: 25 Desquamation or peeling or scabs or scale: 20 Edema: 112 Erosions: 56 Macular or papular: 345 Pink or red: 345 Purplish/purpuric/violaceous: 140 Ulceration: 15 Unspecified rash morphology: 65 Vesicles: 44 | Feet: 364 Hands: 45 Not hands or feet: 10  Dorsal feet: UN  Heels: UN Periungual areas: UN Toes: UN | Pain: 207 Pruritis: 180 Temperature change: 41 Tingling: 23 Unspecified symptoms of rash: 17 |
| Andina D, Noguera-Morel L, *et al.* | Dilated capillaries: UN Erythema: UN Hyperpigmentation: UN Ischemic areas: UN Purpuric dots: UN Violaceous: UN | Blistering: UN Dusky: UN Erythema multiforme: 4 Hyperpigmentation: UN Ischemia: UN Violaceous: UN | Feet: 22 Hands: 3  Fingers: UN Heels: UN  Lateral aspect of feet: UN Toes: UN | Pain: 7 Pruritis: 9 |
| Colonna C, Genovese G, *et al.* |  | Edema: 3 Erythematous-violaceous: 13 Patches or plaques: 13 | Ankles: 2 Dorsum of foot: 4 Feet: 26 Feet & hands: 2  Fingers: 1 Hands: 2 Palm: 1 Palms: 2 Sole of foot: 1 Soles of feet: 9 Toe: 1 Toes: 19 | Pain: 5 Pruritis: 14 |
| Denina M, Pellegrino F, *et al.* |  | Blanching: 24 Blisters: 6 Non-pruriginous: 24 | Feet: 24  Heels: 2 Toes: 22 | Pain: UN |
| Fertitta L, Welfringer-Morin A, *et al.* |  | Chilblain-like lesions: 17 | Feet: 14 Feet & hands: 1 Hands: 2 | Pain: 4 Pruritis: 6 |
| Piccolo V, Neri I, *et al.* |  | Blistering lesion 23  Edema: 31 Erythema: 31 Purpuric: UN | Feet: 85.7% Feet & hands: 7% Hands only: 6% | Asymptomatic: UN  Itch: UN Pain: UN Simultaneous pain and itch: UN |
| Caselli D, Chironna M, *et al.* |  | Blistering: UN Ecchymotic: UN Erosions: UN Erythema: UN Patches: UN Purpuric-ecchymotic patches: UN Red-bluish: UN | Dorsum of hands: UN Feet & hands: UN  Feet: UN Fingers: UN Heel: UN Plantar surface of toes: UN Sole: UN |  |
| Brancaccio G, Gussetti N, *et al.* |  | Bullous: 1 Cyanotic: 1  Ecchymotic: 1 Erythema: 1 Papules: 1 Red: 1  Reddish-purple lesions: 1 | Feet: 2  Plantar surface: 1 Toes: 2 | Pain: 1 |
| Colmenero I, Santonja C, *et al.* |  | Chilblains: 7 | Feet: 7 Feet & hands: 1  Heels: UN Lateral aspects of the feet: UN Toes: UN | Pain: 1 Pruritis: 3 |
| Colonna C, Monzani NA, *et al.* |  | Atrophic lesions: UN  Blurred edges: UN Cyanotic: UN Dusky: UN Erythema: UN Edema: UN  Hyperpigmentation: UN Macules: UN Plaques: UN | Feet: 4 Hand: 1 Dorsal surface of toes: UN Lateral aspect of feet: UN Plantar aspect: UN | Pain: 3 Pruritic: 1 Temperature change (cold): 1 |
| Cordoro KM, Reynolds SD, *et al.* |  | Blisters: UN Dusky: 6 Edema: UN Erythema: UN Hemorrhagic crusts: UN Livedo reticularis: UN Macules: 6 Macules: UN Petechia: UN Plaques: 6 Purpuric: 6 Purpuric: UN Red to violaceous: 6 | Dorsum of foot: UN Feet: 6  Heels: UN Lateral aspect of foot: UN Nailfold area: UN  Soles: UN Toes: 6 | Pruritis: UN |
| Diociaiuti A, Giancristoforo S, *et al.* |  | Crusts: UN Erythemato-violaceous-purpuric: UN Macules: UN Papules: UN Swelling: 17  Onychomadesis: 2 | Feet: 13  Heels: UN Lateral aspect of foot: UN Soles: UN Toes: 17 |  |
| Discepolo V, Catzola A, *et al.* | Desquamation: 2 Pigmented areas: 9 Red dots: 22 Rosettes: 19 Vessel ectasia: 9 White streaks: 17 Winding vessels: 3 | Erythematous, purpuric, or cyanotic: 17 Desquamation: 3 Ulcers: 1 Blurred: UN Erythema: UN Hyperpigmentation: UN Macules: UN Rosaceous: UN | Feet: 15 Feet & hands: 2  Fingers: 2 Heels: 7 Toes: 17 | Pain/ pruritis/ swelling: 9  Burning: 4 |
| Feder HM Jr. |  | Blue: 2 Desquamation: 1 Nodules: 1 Papules: 1 Purple: 1 Red: 3 Swollen: 1 | Feet: 3  Heels: 1 Lateral aspect of foot: 1 Toes: 3 | Pain: 2 Pruritis: 1 |
| Gallizzi R, Sutera D, *et al.* |  | Desquamation: 1 Edema: 9 Erythema: 9 Purple: 9 | Feet: 6 Hands: 3 | Burning: 1 Painful: 2 Pruritic: 3 |
| Garcia-Lara G, Linares-González L, *et al.* |  | Chilblain-like: 25 Erythema multiforme-like: 2 | Feet: 20 Feet & hands: 1 Hands: 6 | Asymptomatic: 18 Pain: 6 Pruritis: 3 |
| Garrido Ruiz MC, Santos-Briz Á, *et al.* |  | Blisters: 1 Crusts: 2 Desquamation: 1 Erythema: 1 Erythematoviolaceous: 2 Erythematous-purpuric: 2 Erythematous-violaceous: 1 Macules: 3 Papules: 1 Plaque: 1 Purple: 1 Swelling: 1 Vesicles: 1 | Dorsum of hands: 1 Dorsum of feet: 1 Dorsum of toes: 3 Feet: 4 Foot: 2 Feet & hands: 1  Fingertips: 1 Heels: 1 Soles of feet: 2 Toes: 1 | Pain: 0 Pruritis: 0 |
| Kerber AA, Soma DB, *et al.* |  | Edema: 1 Erosions: 1 Erythema: 1 Violaceous: 1 Vesicles: 1 | Feet: 1  Plantar aspect of feet: 1 Toes: 1 | Pain: 1 Pruritis: 1 |
| Klimach A, Evans J, *et al.* |  | Erythema: 2 Macules: 1 Papules: 1 Petechiae: 1 | Axillae: 1  Feet: 1 Soles of feet: 1 | Pain: 1 |
| Ladha MA, Dupuis EC |  | Papules: 1 Red-violaceous: 1 | Feet & hand: 1  Finger: 1 Toes: 1 | Pain: 1 |
| Landa N, Mendieta-Eckert M, *et al.* |  | Erythematous: 1 Palpable: 1 Purplish: 1 Purpuric: 1 Reddish: 1 | Fingers: 1 Foot: 1 Foot & hand: 1  Heels: 2 Toes: 1 | Pain: 1 Pruritis: 1 |
| Locatelli AG, Test ER, *et al.* |  | Edema: 1 Erosion: 1 Erythema:1 Macules: 1 Plaques: 1 | Hands & foot: 1  Fingers: 1 Toe: 1 |  |
| Mohan V, Lind R |  | Erythema: 1 Swelling: 1 | Feet: 1  Toes: 1 | Burning: 1 Pruritis: 1 |
| Neri I, Conti F, *et al.* |  | Chilblain: 1 Desquamation: 1 Nodules: 1  Papulopustular: 1 Red-purple: 1 | Feet & hands: 1  Thighs & trunk: 1  Fingertips: UN  Lateral aspect of feet: UN Palms: UN Toes: UN | Pain: 1 |
| Neri I, Patrizi A, *et al.* | Pink center surrounded by a "whitish scale collarette": 5 | Edema: 5 Erythema: 5 Desquamation: UN | Hands: 2 Hands & feet: 3 Palmer surfaces: 5 Plantar surfaces: 3 Fingertips: UN Hypothenar: UN Thenar: UN | Burning: 1 Pain: 1 Pruritis: 5 |
| Neri I, Virdi A, *et al.* |  | Bullae: 3 Nodules: 4 Macules or patches: 8 Purple to red: 12 | Fingers: 3 Feet: 8 Feet & hands: 3  Heels: 3 Soles: 3  Toes: 8 | Pain: 3 Pruritis: 5 Tingling: 1 |
| Nirenberg MS, Herrera MDMR |  | Erythema: 1 | Feet & hand: 1 Fingers: UN Toes: 1 | Pruritic: 1 |
| Rodríguez-Pastor SO, Pedraz L, *et al.* |  | Blister: UN Purpuric: UN | Feet: UN Fingers: UN Hands: UN Heels: UN Lateral aspects of feet: UN Toes: UN | Pain: 44% Pruritis: 26% |
| Papa A, Salzano AM, *et al.* |  | Ulcers: 3 Edema: UN Erythema: UN  Macules: UN  Nodules: UN Plaques: UN Purple to red: UN Vesicles: UN | Lower extremities: 9  Upper extremities: 2 Fingers: UN Hands: UN  Heels: UN Lateral aspect of feet: UN Plantar surface of foot: UN Toes: UN | Allodynia: 10 Burning: 10 Pain: 11 Pruritis: 8 |
| Piccolo V, Bassi A, *et al.* | Brown, round, unstructured area: 1 Bullae: 1 Coppery red background: 6 Crusts: 2 Cuticular vessels: 2 Dotted vessels: 3 Erythematous background: 4 Glomerular vessels: 1 Hemorrhagic dots: 6 Irregular linear vessels: 2 Linear vessels: 2 Multiple short vessels arranged perpendicularly: 1 Purple unstructured area: 3 Vessels with branches: 1 | Blister: 2 Edema: 7 Erythema: 7 | Feet: 7 Hands: 1 Hands & feet: 1 |  |
| Rafai M, Elbenaye J, *et al.* |  | Erythema: 1 Maculopapular: 1 Purpuric: 1 Targetoid: 1 | Feet: 1 Hands: 1  Heels: 1 Palms: 1 Soles: 1 Toes: 1 | Pruritis: 1 |
| Roca-Ginés J, Torres-Navarro I, *et al.* |  | Dactylitis: 5 Erythema: 6 Maculopapules: 8 Purpuric: 8 | Feet: 14 Hands: 2 Hands & feet: 4  Toes: UN |  |
| Rosés-Gibert P, Gimeno Castillo J, *et al.* |  | Erosion: 5 Erythema: 28 Macules: 20 Papules: 28 Purpuric: 20 Swelling: 6 | Dorsum of hands: 2 Dorsum of feet: 21 Feet: 35 Hands: 2 Hands & feet: 1  Heels: 5 Plantar: 4 Toes: 12 | Asymptomatic: 18 Pain: 8 Pruritus: 14 |
| Rouanet J, Lang E, *et al.* |  | Erosion: 3 Erythema: 3 Livedoid: 2 Papules: 2 Patches: 3 Plaques: 1  Post-inflammatory pigmentation: 1 Purple: 3 Scale: 1 | Dorsal surface toes: 3 Feet: 3  Tips of toes: 1 |  |
| Ruggiero G, Arcangeli F, *et al.* |  | Erythema: 1 Bluish-red: 1  Swelling: 10 | Dorsal surface toes: 1 Face: 2  Feet: 20 Foot: 10 Hand: 3 Hands: 2 | Asymptomatic: 17 Burning: 3  Pain: 7 Pruritis: 8 |
| Tammaro A, Adebanjo GAR, *et al.* |  | Pernio-like: 1 | Hand: 1 |  |
| Tosti G, Barisani A, *et al.* |  | Erythema: 3 Papules: 1 Plaques: 2 | Feet: 2  Heels: 2 Toes: 1 | Pain: 2 Pruritis: 1 |
| Vastarella M, Patrì A, *et al.* |  | Erythema: 15 Macules: 15 Papules: 15 Purpuric: 15 Bulli: UN Crusts: UN Digital swelling: UN | Foot: 1 Unspecified location: 14 |  |
| Hubiche T, Phan A, *et al.* |  | Acrocyanosis: 13 Acrocholosis: 3 Acrorhigosis: 7 Cheilitis: 1 Chilblain-like: 82 Eccrine hidradenitis-like: 6 Edema: 14 Erythema: 52  Erythema multiforme-like: 3 Erythema nodosa: 2 Hyperhidrosis: 10 Livedo: 6 Maculopapular: 3 Pulpitis: 7 Papules: 2 Purpura: 9 Telangiectasia: 4 Urticaria: 3 Vesicles: 19 | Feet: 16 Hands: 15 Hands & feet: 71 | Pain: 50 Pruritus: 58 |
| L. Rizzoli, L. Collini, *et al.* |  | Chilblains-like lesions: 11 | Feet: 9 Hands: 3 |  |
| Recalcati S, Gianotti R, *et al.* |  | Chilblains-like lesions: 2 | Feet & hands: 2 | Pain: 1 |
| El Hachem M, Diociaiuti A, *et al.* | Abnormal capillary morphology: 10 Enlarged capillary dimension: 7 Microhemorrhages: 7 Pericapillary edema: 5 Reduced capillary density: 1 | Crusts: 12 Erythema: 18 Macules: 13 Papules: 1 Pustules: 3 Purpura or violaceous: 13 Swelling: 16 Erosions: UN | Feet: 19  Heels: 8 Heel: 2 Soles of feet: 8 Toes: 19 | Asymptomatic: 8 Burning: 2 Pain: 5 Pruritis: 7 |
| Herman A﻿, Peeters  C﻿, *et al.* |  | Purplish-red: 14 | Foot: 13 Foot & hand: 1 |  |
| Kluckow E, Krieser DM, *et al.* |  | Erythema: 4 Swelling: 3 | Feet: 2 Fingers: 3 Foot: 1 Hand: 3  Toes: 3 | Pain: 1 Pruritis: 2 |
| Fabbrocini G, Vastarella M, *et al.* | Enlarged vessels: 8 Dotted vessels: 23 Follicular red dots: 7 Melanin hyperpigmentation: 7 Rosettes: 12 Scale: 8 White streaks: 8 Wickham striae: 1 Yellow structureless area: 6 | Bulli: UN Crusts: UN Digital swelling: UN Erythema: UN Macules: UN Papules: UN Post-inflammatory hyperpigmentation: UN Purpuric: UN Rosaceous: UN Scale: UN | Ankle: 1 Feet: 14 Feet & hands: 1  Heels: 6 Toes: 15 | Burning: UN Pruritis: UN |
| Colonna C, Spinelli F, *et al.* |  | Edema: UN Macules: UN Plaques: UN Dusky to purpuric: UN Red to violaceous: UN | Feet: 4  Heels: UN Lateral margin of feet: UN Soles of the feet: UN | Pain: UN |
| Magro CM, Mulvey JJ, *et al.* |  | Pernio-like: 1 Targetoid: 1 Ulceration: 1 | Feet: 1 Toes: 1 |  |
| Jacquin-Porretaz C, Ducournau A, *et al.* |  | Pseudo-chilblain: 7 | Location of lesions not specified: 7 |  |
| Recalcati S, Tonolo S, *et al.* |  | Chilblains-like: 2 | Feet: 12 Feet & hands: 9 Hands: 4 | Itch: 2 Pain: 2 |

* Only studies that recorded the number of cases were included in counts (studies reporting percentages were excluded from counts)

^Counts of hand, hands, foot, feet, and hand & foot/hands & foot/hand & feet/hands & feet are included for each study if available; if more specific locations or different locations were available, they were recorded

UN: unspecified number
