# Supplementary material for: Chilblains-Like Lesions in Pediatric Patients: A Review of Their Epidemiology, Etiology, Outcomes, and Treatment
Source: Front Pediatr. 2022 Jun 23;10:904616. doi: 10.3389/fped.2022.904616 (PMC9259963; doi:10.3389/fped.2022.904616)
Supplement: Supplementary file 2 [file Table_2.DOCX]

Table S2. Biopsy Results & Lesion Treatments

| Study | Histopathology | Lesion Treatment |
| --- | --- | --- |
| Castelo-Soccio L, Lara-Corrales I, *et al.* | C3: 3 Deep lymphocytic infiltrate: 12 Deep perieccrine inflammation: 6 Dermis edema: 6 Eosinophils: 0 Fibrinogen: 3 Fibrinoid change: 2 IgM: 3 Lymphocytic vasculitis: 3 Necrotic keratinocytes (epidermis): 1 Spongiosis: 1 Superficial lymphocytic infiltrate: 12 Vacuolation of basal layer: 8 | Aspirin: 11 Nifedipine cream: 1 NSAIDs: 24 Other treatment: 57 Topical steroids: 145 Warming measures 74 |
| Andina D, Noguera-Morel L, *et al.* | Deep lymphocytic infiltrate (angiocentric): UN Deep lymphocytic infiltrate (eccrinotropic): UN Edema (papillary dermal): UN  Focal thrombosis (papillary dermal vessels): UN Focal thrombosis (reticular dermis): UN Lymphocytic eccrine hidradenitis: UN Lymphocytic exocytosis (acrosyringia) : UN Lymphocytic exocytosis (epidermis): UN Mucinosis (dermal): UN Mucinosis (perieccrine): UN Red cell extravasation: UN Superficial lymphocytic infiltrate (angiocentric): UN Superficial lymphocytic infiltrate (eccrinotropic): UN Vacular degeneration (basal layer): UN Vascular ectasia: UN | Oral analgesics: UN Oral antihistamines: UN Topical corticosteroids & oral steroids (tx for erythema multiforme): 4 |
| Colonna C, Genovese G, *et al.* | Lymphocytic infiltrate (periadnexal dermal): 2 Lymphocytic infiltrate (perivascular dermal): 2 |  |
| Denina M, Pellegrino F, *et al.* |  | Oral corticosteroids: 4  Topical corticosteroids: 9 |
| Brancaccio G, Gussetti N, *et al.* | Endothelial hyperplasia: 1 Lymphocytic infiltrate of wall: 1 Lymphocytic vasculitis: 1 |  |
| Colmenero I, Santonja C, *et al.* | Basal membrane thickening: 0 CD3+ and CD20+ inflammatory cells: 7 CD3+, CD20+, CD79+, CD30+: 1 CD3+, CD20+, CD79a+: 4 CD4+ predominance: 6 CD8+ cells present, less populous than CD4+ cells Dermal edema: 7 Dermal mucin: 5 Erosion/ulceration: 0 Exocytosis: 3 Fibrinoid necrosis: 2 Lymphocytic infiltration of vessels: 7 Lymphocytic panniculitis: 6  Necrotic keratinocytes: 4 Panniculitis: 6 Parakeratosis: 4 Perieccrine inflammation: 6 Perivascular inflammation (deep): 7 Perivascular inflammation (superficial): 7 Purpura:7 SARS-CoV-2 spike protein positive on immunohistology: 7 Spongiosis: 7 Subcutaneous lymphocytic vasculitis: 1 Thrombi in deep dermis: 4 Thrombi in superficial dermis: 1 Thrombi on CD61: 4 Vacuolar changes (basal): 7 Vascular ectasia: 3 On electron microscopy, membrane bound structures noted and recorded as "coronavirus-like particles" and tubulo-reticular inclusions were also noted |  |
| Colonna C, Monzani NA, *et al.* | Endothelial cell swelling: 1 Fibrin thrombus (superficial capillaries): 1 Lymphocytic infiltration (periadnexal): 1 Lymphocytic infiltration (perivascular): 1 Red blood cell extravasation: 1 | No treatment: 4 |
| Cordoro KM, Reynolds SD, *et al.* | Deep lymphocytic infiltrate (perieccrine): UN Deep lymphocytic infiltrate (perivascular): UN DIF negative: 6 Hemorrhagic parakeratosis (stratum corneum): UN Lymphocytes (small vessel walls): UN Purpura: UN Superficial lymphocytic infiltrate (perieccrine): UN Superficial lymphocytic infiltrate (perivascular): UN Vacuolar change: UN |  |
| Discepolo V, Catzola A, *et al.* | Endothelial hyperplasia: 11 Increased blood vessels: 9 Lymphocytic infiltrate (perivascular): 11 No eosinophils: 1 article No neutrophils: 1 article SARS-CoV-2 negative (in situ hybridization): 12 Vacuolar changes: 1 article | Topical antibiotics and/or corticosteroids (patient initiated): 4 Disinfectants (patient initiated): 4 Antifungal treatments (patient initiated): 3 Systemic antibiotics or corticosteroids (patient initiated): 3 |
| Feder HM Jr. |  | Indomethacin: 1 Nifedipine: 1 |
| Gallizzi R, Sutera D, *et al.* |  | No treatment: 9 |
| Garcia-Lara G, Linares-González L, *et al.* |  | No treatment: 27 |
| Garrido Ruiz MC, Santos-Briz Á, *et al.* | C1q+: 6 C3d+: 5 C4d: 6 CD61+: 6 Chilblain-like with no thrombotic vasculopathy: 3  Chilblain-like with thrombotic vasculopathy: 1 Dermatitis: 1 Electron microscopy showed viral particles: UN Endothelitis: 1 Leukocytoclastia: 1 Necrosis of epidermis: 1 Neutrophils: 1 Thrombogenic vasculitis: 1 Thrombotic vasculopathy without vasculitis: 1 Thrombotic vasculopathy: 1 |  |
| Ladha MA, Dupuis EC | Deep lymphocytic inflammation (dermis): 1 Superficial lymphocytic inflammation (dermis): 1 Subepidermal edema: 1 | Avoid cold/damp: 1 Topical corticosteroids: 1 |
| Landa N, Mendieta-Eckert M, *et al.* |  | Azithromycin: 1 Hydroxychloroquine: 1 Prophylactic heparin: 1 |
| Locatelli AG, Test ER, *et al.* | Deep lymphocytic infiltrate (perieccrine): 1 Deep lymphocytic infiltrate (perivascular): 1 Edema (papillary dermis): 1 Superficial lymphocytic infiltrate (perieccrine): 1 Superficial lymphocytic infiltrate (perivascular): 1 |  |
| Mohan V, Lind R |  | Topical glucocorticoid cream: 1 |
| Neri I, Conti F, *et al.* | Deep dermatitis (perivascular): 1 Edema: 1 Endothelialitis: 1 Perivascular lymphocytic infiltrate: 1 SARS-CoV-2 PCR negative: 1 SARS-CoV-2 PCR positive: 1 Superficial dermatitis (perivascular): 1  Thick-walled vessels: 1 |  |
| Neri I, Virdi A, *et al.* | CD3+ T cells: 2 Deep dermatitis (perivascular, interface, vacuolar, & focal lichenoid): 2 Dermoepidermal junction with lichenoid infiltrate of lymphocytes: 2 Dilated vessels (papillary dermis): 2 Edema (papillary dermis): 2 Lymphocytes (papillary dermis): 2  Lymphocytes (perivascular, dermis): 2 Necrotic keratinocytes (epidermis): 2 Superficial dermatitis (perivascular, interface, vacuolar, & focal lichenoid): 2 | Avoid cold exposure: 5 Topical steroids: 5 |
| Nirenberg MS, Herrera MDMR |  | Topical hydrocortisone 1%: 1 |
| Papa A, Salzano AM, *et al.* |  | Paracetamol 15 mg/kg (max 750 mg) PO every 6-8 hours for 10 days: 11 |
| Roca-Ginés J, Torres-Navarro I, *et al.* | Dermal thrombi: 2 Edema: 3 Endothelial swelling: 6 Fibrin deposition: 3 Infiltrate in papillary dermis: 6 Lymphocytic vasculitis: 3 Necrotic keratinocytes: 6 Perieccrine infiltrate: 6 Perivascular infiltrate: 6 Spongiosis: 6 Vacuolar changes: 6 |  |
| Rosés-Gibert P, Gimeno Castillo J, *et al.* |  | Topical steroids or antibiotics: 6 |
| Rouanet J, Lang E, *et al.* | SARS-CoV-2 PCR negative: 3 |  |
| Ruggiero G, Arcangeli F, *et al.* |  | Antibiotics: number not specified Antihistamines: number not specified Heparin gel: 1 Mometasone furoate cream: 1 Paracetamol: number not specified Topical steroids: number not specified |
| Tammaro A, Adebanjo GAR, et al. | C4d (endothelium): 1 CD8+ predominance (endothelium): 1 Endothelial cell degeneration: 1 Fibrin (luminal): 1 Fibrin: 1  Lymphocytes (perivascular zone): 1 Lymphocytes (perivascular): 1 Lymphocytes (tissue): 1 Necrosis (focal): 1 Neutrophils (perivascular zone): 1 Neutrophils (tissue): 1 Red blood cell extravasation: 1 SARS-CoV-2 PCR positive: 1 Thickened vascular walls: 1 Vessel thrombosis: 1 |  |
| Tosti G, Barisani A, *et al.* |  | Emollients: 2 Paracetamol: 2 |
| El Hachem M, Diociaiuti A, *et al.* | Apoptotic keratinocytes: 4 Edema (papillary dermis): 12 Extravasation of erythrocytes: 15 Fibrin thrombi: 2 Lymphocytic exocytosis: 6 Lymphocytic infiltrate (perieccrine): 18 Lymphocytic infiltrate (perivascular): 18 Lymphocytic vasculitis: 3 Mucin: 17 SARS-CoV-2 PCR negative: 3 Spongiosis: 13 Vacuolation (basal epidermal layer): 14 | No treatment: 19 |
| Herman A﻿, Peeters  C﻿, *et al.* | C3: 5 IgA: 1 IgM: 2 Lymphocytic infiltrate (perisudoral): 6 Lymphocytic infiltrate (perivascular): 10 Lymphocytic vasculitis: 1 Microthrombosis: 4 SARS-CoV-2 PCR negative: 10 Vacuolar alteration (perisudoral): 1 |  |
| Magro CM, Mulvey JJ, *et al.* | Deep histiocytic infiltrate (perieccrine): 1 Deep histiocytic infiltrate (perivascular): 1 Deep hystiocytic infiltrate (intramural): 1 Deep lymphocytic infiltrate (intramural): 1 Deep lymphocytic infiltrate (perieccrine): 1 Deep lymphocytic infiltrate (perivascular): 1 Edema (papillary dermal): UN: 1 Endothelial swelling: 1 Extravasation of erythrocytes: 1 Fibrin (blood vessels): 1 Interface dermatitis: 1 Superficial histiocytic infiltrate (perieccrine): 1 Superficial histiocytic infiltrate (perivascular): 1 Superficial hystiocytic infiltrate (intramural): 1 Superficial lymphocytic infiltrate (intramural): 1 Superficial lymphocytic infiltrate (perieccrine): 1 Superficial lymphocytic infiltrate (perivascular): 1 |  |
| Recalcati S, Tonolo S, *et al.* |  | No treatment: UN |

UN: unspecified number
