# Supplementary material for: Chilblains-Like Lesions in Pediatric Patients: A Review of Their Epidemiology, Etiology, Outcomes, and Treatment
Source: Front Pediatr. 2022 Jun 23;10:904616. doi: 10.3389/fped.2022.904616 (PMC9259963; doi:10.3389/fped.2022.904616)
Supplement: Supplementary file 3 [file Table_3.DOCX]

Table S3. SARS-CoV-2 Exposure & Systemic Symptoms of Illness

| Study | Contact with Possible SARS-CoV-2 Casesᶲ | Contact with Positive SARS-CoV-2 Cases | Systemic Symptoms of Cases |
| --- | --- | --- | --- |
| Castelo-Soccio L, Lara-Corrales I, *et al.* | 133 | 10 | Abdominal symptoms: 16 Anosmia: 2 Asymptomatic: 264 Chest pain: 1 Cough: 44 Fever: 48 Irritable: 1 Malaise: 18 Myalgias: 10 Other mucous: 2 Other: 46 Rhinorrhea: 32 Sore throat: 25 Ulcers: 1 |
| Andina D, Noguera-Morel L, *et al.* | 12 | 1 | Abdominal symptoms or diarrhea: 2 Cough or rhinorrhea: 9 Fever: 0 Short of breath: 0 |
| Colonna C, Genovese G, *et al.* | 7 | 3 | Abdominal symptoms: 1 Coryza: 3 Cough: 6 Dyspnea: 2 Fever: 9 Headaches: 1 Pharynx pain: 2 Weakness: 2 |
| Denina M, Pellegrino F, *et al.* | 7 | 2 | Conjunctivitis: 3 Cough: 10 Fever: 4 Gastrointestinal symptoms: 5 |
| Fertitta L, Welfringer-Morin A, *et al.* | 14 | 2 | Anosmia: 0 Articular symptoms: 0 Asymptomatic: 7 Digestive symptoms: 3 ENT symptoms: 7 Fever: 3 Influenza-like symptoms: 7 Respiratory symptoms: 6 |
| Piccolo V, Neri I, *et al.* | 8 | 2 | Fever: UN Gastrointestinal symptoms: UN Respiratory symptoms: UN |
| Caselli D, Chironna M, *et al.* |  | 1 | Diarrhea: 2 Fever: 6 |
| Brancaccio G, Gussetti N, *et al.* |  | 1 | Asymptomatic: 1  Symptoms of SARS-CoV-2: 1 |
| Colmenero I, Santonja C, *et al.* | 4 |  | Gastrointestinal symptoms: 1 Respiratory symptoms: 5 |
| Colonna C, Monzani NA, *et al.* | 3 |  | Cough: 1 Edema: 1 Fever: 2 Headache: 1 Influenza-like illness: 1 Pain: 3 Rhinitis: 1 |
| Cordoro KM, Reynolds SD, *et al.* | 6 | 0 | Asymptomatic: 4 Congestion: 2 Fever: 2 Rhinorrhea: 2 Sore throat: 2 |
| Diociaiuti A, Giancristoforo S, *et al.* | 2 | 1 | Asthenia: 1 Asymptomatic: 10 Headache: 1 Influenza-like symptoms: 2 |
| Discepolo V, Catzola A, *et al.* | 2 | 2 | Asymptomatic: 12 Cough: 1 Diarrhea: 1 Dysgeusia: 1  Gastrointestinal symptoms: 1 Rhinitis: 1 Sore throat: 2 Upper respiratory tract infection: 4 |
| Feder HM Jr. |  | 2 | Asymptomatic: 3 |
| Gallizzi R, Sutera D, *et al.* | 0 | 2 | Asymptomatic: 3 Asthenia: 1 Chest pain: 1 Chills: 1 Dyspnea: 1 Fever: 4 Nasal congestion: 1 |
| Garcia-Lara G, Linares-González L, *et al.* | 0 | 7 | Diarrhea: UN |
| Garrido Ruiz MC, Santos-Briz Á, *et al.* |  |  | Asymptomatic: 5 Diarrhea: 1 Fever: 1 |
| Kerber AA, Soma DB, *et al.* |  |  | Cough: 1 Fever: 1 Pharyngitis: 1 |
| Klimach A, Evans J, *et al.* |  | 2 | Fever: 1 Headache: 1 Myalgia: 1 |
| Ladha MA, Dupuis EC |  |  | Asymptomatic: 1 |
| Landa N, Mendieta-Eckert M, *et al.* |  | 1 | Asymptomatic but found to have bilateral pneumonia: 1 Diarrhea: 1 Nasal congestion: 1 |
| Locatelli AG, Test ER, *et al.* |  | 1 | Diarrhea: 1 Dysgeusia: 1 |
| Mohan V, Lind R |  |  | Cough: 1 Fatigue: 1 Fever: 1 Myalgias: 1 |
| Neri I, Conti F, *et al.* |  | 1 | Asthenia: 2 Fever: 1 |
| Neri I, Patrizi A, *et al.* |  |  | Fever: 2 Maculopapular rash: 2 |
| Neri I, Virdi A, *et al.* | 0 | 0 |  |
| Nirenberg MS, Herrera MDMR | 1 |  | Back pain: 1 Diarrhea: 1 Headache: 1 Rash on abdomen: 1  Rash on chest: 1 Rash on face: 1 Sore throat: 1 |
| Rodríguez-Pastor SO, Pedraz L, *et al.* | 3 | 1 | Abdominal pain: 4 Cough: 2 Diarrhea: 7 Emesis: 2 Fever: 4 Headache: 1 Myalgias: 1 Respiratory distress: 0 Sore throat: 3 |
| Papa A, Salzano AM, *et al.* |  |  | Bilateral conjunctivitis: 4 Cough: 2 Fever: 2 |
| Rafai M, Elbenaye J, *et al.* |  |  | Clinical symptoms of SARS-CoV-2: 1 |
| Roca-Ginés J, Torres-Navarro I, *et al.* |  |  | Asymptomatic: 20 |
| Rosés-Gibert P, Gimeno Castillo J, *et al.* | 12§ |  | Asthenia: 2 Cough: 7 Diarrhea: 3 Fever: 3 Myalgia: 2 Nausea: 3 Vomiting: 3 |
| Rouanet J, Lang E, *et al.* |  | 0 | Arthralgia: 1 Asthenia: 2 Cough: 1 Headache: 2 Myalgia: 1 |
| Ruggiero G, Arcangeli F, *et al.* |  |  | Cough: 1 Erythema multiforme: 1 Fever: 1 Sore throat: 1 |
| Tammaro A, Adebanjo GAR, *et al.* |  |  | Asymptomatic: 1 |
| Tosti G, Barisani A, *et al.* |  |  | Asymptomatic: 1 Pharyngodynia: 1 |
| Vastarella M, Patrì A, *et al.* |  | 3 | SARS-CoV-2-like symptoms: 6 |
| Hubiche T, Phan A, *et al.* |  | 66 | Fever: 9 |
| L. Rizzoli, L. Collini, *et al.* |  | 2 |  |
| Recalcati S, Gianotti R, *et al.* |  |  | Ageusia: 1 Anosmia: 1 Fever: 1 Headache: 1 |
| El Hachem M, Diociaiuti A, *et al.* | 7 | 0 | Cough: 1 Diarrhea: 1 Fever: 5 Headache: 1 Sore throat: 2 |
| Herman A﻿, Peeters  C﻿, *et al.* | 1§ |  | Asymptomatic: 5 Conjunctivitis: 1 Diarrhea: 2 Dyspnea: 2 Rhinitis: 7 Sore throat: 2 |
| Kluckow E, Krieser DM, *et al.* | 0 | 0 | Asymptomatic: 3 Cough: 1 |
| Fabbrocini G, Vastarella M, *et al.* |  | 3 | SARS-CoV-2-like symptoms: 6 |
| Colonna C, Spinelli F, *et al.* | UN |  | Cough: UN Gastrointestinal disorders: UN |
| Magro CM, Mulvey JJ, *et al.* | 1 |  |  |
| Recalcati S, Tonolo S, *et al.* |  |  | Ageusia: 1 Anosmia: 1 Asymptomatic: 15 Cold: 1 Cough: 1 Diarrhea: 1 Fever: 6 Headache: 2 |

ᶲ If contacts had symptoms, but tested negative for SARS-CoV-2, they were not considered a possible contact

§ Possible infection and confirmed SARS-CoV-2 reported together

UN: unspecified number
