# Supplementary material for: Chilblains-Like Lesions in Pediatric Patients: A Review of Their Epidemiology, Etiology, Outcomes, and Treatment
Source: Front Pediatr. 2022 Jun 23;10:904616. doi: 10.3389/fped.2022.904616 (PMC9259963; doi:10.3389/fped.2022.904616)
Supplement: Supplementary file 4 [file Table_4.DOCX]

Table S4. Acute Infections, Past Medical Histories, & Medications of Cases*

| Study | Acute Infections | Past Medical History of Cases | Medications |
| --- | --- | --- | --- |
| Castelo-Soccio L, Lara-Corrales I, *et al.* |  | Sjögren disease: 1 Unspecified comorbidities: 18.3% |  |
| Andina D, Noguera-Morel L, *et al.* |  | Acrocyanosis: 0 Attention-deficit hyperactivity disorder: 5 Chilblains: 0  Raynaud phenomenon: 0 Rheumatic disease: 0 Systemic lupus erythematosus: 0 | Methylphenidate hydrochloride: 3  Methylphenidate hydrochloride, aripiprazole, & intuniv: 1 lisdexamfetamine: 1 |
| Colonna C, Genovese G, *et al.* |  |  | No new medications within 15 days of lesion onset: 30 |
| Denina M, Pellegrino F, *et al.* |  | Chilblains: 0 | Methylphenidate hydrochloride: 2 |
| Fertitta L, Welfringer-Morin A, *et al.* |  | Asthma: 3 Atopic dermatitis: 2 Auto-immune disease: 0 Inflammatory Bowel Disease: 0 Obesity: 0 Photosensitivity: 0 Raynaud phenomenon: 0 Urticaria: 1 |  |
| Piccolo V, Neri I, *et al.* | *Mycoplasma pneumonia positive*: 1 | ANA positive: 1 Autoimmune disorders: 5 Coagulation disorders: 4‡ Drug allergy: 1 Peripheral neuropathy: 1 Wolff-Parkinson-White: 1 |  |
| Caselli D, Chironna M, *et al.* | Adenovirus positive: 0 Bocavirus positive: 0 *Bordetella parapertussis* positive: 0 *Bordetella pertussis* positive: 0 *Chlamydophila pneumoniae* positive: 0 Enterovirus positive: 0 *Haemophilus influenzae* positive: 0 Human Coronavirus OC43, NL63, 229E positive: 0 Human Rhinovirus positive: 0 Influenza A positive: 0 Influenza B positive: 0 *Legionella pneumophila* positive: 0 Metapneumovirus positive: 0 *Mycoplasma pneumonia* positive: 1 Parainfluenza ½/3/4 positive: 0 Respiratory Syncytial virus A/B positive: 0 *Streptococcus pneumoniae* positive: 0 | Autoimmune disorders: 5 ANA positive: 1 Celiac disease: 2 Coagulation defects: 4‡ Diabetes: 1 Nephrotic syndrome: 1 X-linked disease: 1 |  |
| Colmenero I, Santonja C, *et al.* |  | Acrocyanosis: 0 Attention-deficit hyperactivity disorder: 2  Prior chilblains: 0 Raynaud phenomenon: 0 Rheumatic disease: 0 SLE: 0 |  |
| Colonna C, Monzani NA, *et al.* | Cytomegalovirus positive: 0 Epstein-Barr virus positive: 0 *Mycoplasma pneumonia* positive: 0 Parvovirus B19 positive: 0 | No medical conditions: 1 |  |
| Cordoro KM, Reynolds SD, *et al.* |  | No medical conditions: 6 Coagulation disorders: 0 |  |
| Discepolo V, Catzola A, *et al.* |  | Idiopathic perniosis: 0 IgA vasculitis: 1 Raynaud phenomenon: 0 |  |
| Feder HM Jr. | Epstein-Barr virus positive: 0 Parvovirus B19 positive: 0 |  |  |
| Gallizzi R, Sutera D, *et al.* |  | Acrocyanosis: 0 Autoimmune disorders: 0 Chilblains: 0 Photosensitivity: 0 Raynaud phenomenon: 1 |  |
| Garcia-Lara G, Linares-González L, *et al.* |  | Chilblains: 0 Raynaud phenomenon: 0 |  |
| Kerber AA, Soma DB, *et al.* | Epstein-Barr virus positive: 0 Influenza positive: 0 Streptococcal positive: 0 |  |  |
| Klimach A, Evans J, *et al.* | Adenovirus positive: 0 Enterovirus positive: 0 Human metapneumovirus positive: 0 Influenza A positive: 0 Influenza B positive: 0 *Mycoplasma pneumonia* positive: 0 Parainfluenza positive: 0 Respiratory syncytial virus positive: 0 Rhinovirus positive: 0 Seasonal corona virus positive: 0 | No medical conditions: 1 |  |
| Landa N, Mendieta-Eckert M, *et al.* |  | Asthma: 1 |  |
| Locatelli AG, Test ER, *et al.* |  | Autoimmune disorders: 0 Raynaud phenomenon: 0 |  |
| Mohan V, Lind R |  | Seasonal allergies: 1 | Not on medication: 1 |
| Neri I, Patrizi A, *et al.* | Cytomegalovirus positive: 0 Epstein-Barr virus positive: 1 Enterovirus positive: 0 Group A beta-hemolytic streptococci positive: 0 *Mycoplasma pneumonia* positive: 0 Parvovirus B19 positive: 0 |  |  |
| Neri I, Virdi A, *et al.* | Cytomegalovirus positive: 0 Epstein-Barr virus positive: 0 Enterovirus positive: 0 *Mycoplasma pneumonia* positive: 0 Parvovirus positive: 0 |  |  |
| Nirenberg MS, Herrera MDMR | Chlamydia positive: 0 Cytomegalovirus positive: 0 Epstein-Barr virus positive: 0 Hepatitis B positive: 0 Hepatitis C positive: 0 *Mycoplasma pneumonia* positive: 0 Parvovirus B19 positive: 0 Syphilis positive: 0 | No medical issues: 1 |  |
| Rodríguez-Pastor SO, Pedraz L, *et al.* | *Chlamydia pneumoniae* positive: 1 Cytomegalovirus IgG positive: 10 Epstein-Barr virus IgG positive: 24 Epstein-Barr virus IgM positive: 3 HSV-1 IgG positive: 7 Parvovirus B19 IgG positive: 8 Parvovirus B19 IgM positive: 1 |  |  |
| Papa A, Salzano AM, *et al.* |  | Acrocyanosis: 0 Raynaud phenomenon: 0 |  |
| Piccolo V, Bassi A, *et al.* |  | No medical problems: 9 |  |
| Rafai M, Elbenaye J, *et al.* |  | No medical problems: 1 |  |
| Roca-Ginés J, Torres-Navarro I, *et al.* |  | Raynaud phenomenon or perniosis: 9 Systemic Lupus Erythematosus: 1 | Acetaminophen: 1 Ferric sulfate: 1 |
| Rouanet J, Lang E, *et al.* |  | Chilblains: 0 Raynaud phenomenon: 0 |  |
| Tosti G, Barisani A, *et al.* |  | Alopecia areata universalis: 1 Chilblains: 0 No medical problems: 1 Raynaud phenomenon: 0 |  |
| Vastarella M, Patrì A, *et al.* | Infection positive: 0 | Autoimmune conditions: 0 |  |
| L. Rizzoli, L. Collini, *et al.* | Cytomegalovirus positive: 0 Epstein-Barr virus positive: 0 Enterovirus IgM undefined and IgG negative: 1 Parvovirus B19 positive: 0 | Chilblains: 0 Rheumatic disease: 0 |  |
| Recalcati S, Gianotti R, *et al.* | Cytomegalovirus: 0  Coxsackie: 0 Epstein-Barr virus: 0 Parvovirus B19: 0 |  |  |
| El Hachem M, Diociaiuti A, *et al.* | Parvovirus B19: 0 |  |  |
| Herman A﻿, Peeters  C﻿, *et al.* |  | Allergic rhinoconjunctivitis: 1 Chilblains: 2 Crohn disease: 1 Headache: 1 Kawasaki disease: 1 Raynaud phenomenon: 0 |  |
| Kluckow E, Krieser DM, *et al.* |  | ADHD: 1 Chilblains or related conditions: 0 |  |
| Fabbrocini G, Vastarella M, *et al.* | Epstein-Barr virus IgM positive: 1 | Autoimmune disease: 0 |  |

‡ Coagulation defects in cases and family reported together as 4

* Only studies that recorded the number of cases were included in counts (studies reporting percentages were excluded from counts)
