# Supplementary material for: Chilblains-Like Lesions in Pediatric Patients: A Review of Their Epidemiology, Etiology, Outcomes, and Treatment
Source: Front Pediatr. 2022 Jun 23;10:904616. doi: 10.3389/fped.2022.904616 (PMC9259963; doi:10.3389/fped.2022.904616)
Supplement: Supplementary file 5 [file Table_5.DOCX]

Table S5. Time between Systemic Symptoms Onset and Lesion Onset, & Environmental Exposures

| Study | Latency between Systemic Symptoms and Lesions | Environmental Exposures |
| --- | --- | --- |
| Andina D, Noguera-Morel L, *et al.* | 0-7 days: 4 7-14 days: 2 14-28 days: 4 |  |
| Colonna C, Genovese G, *et al.* | 6 days (median) with a range of -1 to 35 days: 13 |  |
| Fertitta L, Welfringer-Morin A, *et al.* | 22 days (mean) with a range of 5-46 days: 6 Systemic symptoms appeared 19 days after lesion onset: 2 Simultaneous onset: 2 |  |
| Piccolo V, Neri I, *et al.* | Systemic symptoms preceded lesions in most cases: UN |  |
| Caselli D, Chironna M, *et al.* | 1 month: 8 |  |
| Brancaccio G, Gussetti N, *et al.* | 21 days:1 |  |
| Colonna C, Monzani NA, *et al.* | 4 days: 1 10 days: 1 20 days:1 Systemic symptoms appeared several weeks before lesions: 1 |  |
| Cordoro KM, Reynolds SD, *et al.* | 7 days: 2 |  |
| Diociaiuti A, Giancristoforo S, *et al.* | 1 month: 3 |  |
| Discepolo V, Catzola A, *et al.* | 3 weeks: 5 |  |
| Gallizzi R, Sutera D, *et al.* | 4 days: 1 1 week: 1 3 weeks: 3 Simultaneous: 1 |  |
| Garcia-Lara G, Linares-González L, *et al.* | Simultaneous: 1 |  |
| Garrido Ruiz MC, Santos-Briz Á, *et al.* | Systemic symptoms appeared 1 week after lesion onset: 1 |  |
| Kerber AA, Soma DB, *et al.* | 6 weeks: 1 |  |
| Klimach A, Evans J, *et al.* | Simultaneous: 1 |  |
| Landa N, Mendieta-Eckert M, *et al.* | 1 week: 1 |  |
| Locatelli AG, Test ER, *et al.* | 3 days: 1 |  |
| Mohan V, Lind R | 2 weeks: 1 |  |
| Neri I, Conti F, *et al.* | Simultaneous: 2 |  |
| Neri I, Virdi A, *et al.* |  | Barefoot or thin socks: 8 Cold floors: 7 Home heating not on: 5 |
| Nirenberg MS, Herrera MDMR | 4 days: 1 |  |
| Roca-Ginés J, Torres-Navarro I, *et al.* |  | Not wearing shoes in home: 15 Barefoot walking in home: 15 Heating not available in home: 18 |
| Rosés-Gibert P, Gimeno Castillo J, *et al.* | 12.62 days (mean): 8 Simultaneous or overlapping appearance: 3 |  |
| Rouanet J, Lang E, *et al.* |  | Heated homes: 10 |
| Tosti G, Barisani A, *et al.* | 2 weeks: 1 |  |
| Recalcati S, Gianotti R, *et al.* | 21 days: 1 |  |
| El Hachem M, Diociaiuti A, *et al.* | 1 month: 1 1.5 months: 1 2 months: 3  Systemic symptoms appeared 1 week after lesion onset: 1 | Barefoot walking: 11 Wearing socks while walking: 5 |
| Herman A﻿, Peeters  C﻿, *et al.* |  | Decrease in physical activity: 13 No change in physical activity: 1 Increase in screen time: 14 Routine shoe wearing: 1 |
| Kluckow E, Krieser DM, *et al.* |  | No cold exposure: 4 |
| Fabbrocini G, Vastarella M, *et al.* | Within 4 months: 6 | No cold exposure: 15 |
| Recalcati S, Tonolo S, *et al.* | 7 days: 1 10 days: 2 14 days: 1 15 days: 1 21 days: 3 42 days: 1 56 days: 1 |  |

UN: unspecified number
