# Supplementary material for: Chilblains-Like Lesions in Pediatric Patients: A Review of Their Epidemiology, Etiology, Outcomes, and Treatment
Source: Front Pediatr. 2022 Jun 23;10:904616. doi: 10.3389/fped.2022.904616 (PMC9259963; doi:10.3389/fped.2022.904616)
Supplement: Supplementary file 6 [file Table_6.DOCX]

Table S6. Family History of Cases, & Family Members with Lesions

| Study | Family History of Cases | Family Members with Lesions |
| --- | --- | --- |
| Castelo-Soccio L, Lara-Corrales I, *et al.* | Autoimmune history negative: 34 |  |
| Andina D, Noguera-Morel L, *et al.* |  | 2 cases were brothers |
| Piccolo V, Neri I, *et al.* | Coagulation defects: 4‡ |  |
| Caselli D, Chironna M, *et al.* | Coagulation defects: 4‡ |  |
| Cordoro KM, Reynolds SD, *et al.* | Autoimmune genetic disorders: 0 Coagulation disorders: 0 Inflammatory disorders: 0 | Cases were 2 sets of 3 siblings from 2 unrelated families |
| Discepolo V, Catzola A, *et al.* | Autoimmune thyroiditis: 5 Hemolytic anemia during pregnancy: 2 Inflammatory bowel disease: 1 Thromboangiitis obliterans: 1 |  |
| Feder HM Jr. |  | Cases were 3 siblings |
| Gallizzi R, Sutera D, *et al.* | Acrocyanosis: 0 Autoimmune disorders: 0 Chilblains: 0 Photosensitivity: 0 Raynaud phenomenon: 0 | 2 cases were siblings |
| Neri I, Patrizi A, *et al.* | Atopy: 4 Chilblain: 1 Dyshidrosis: 1 Hyperhydrosis: 1 |  |
| Neri I, Virdi A, *et al.* | Chilblains: 2 Connective autoimmune disease: 0 |  |
| Rodríguez-Pastor SO, Pedraz L, *et al.* | Ischemic disease: 0 Vascular disease: 0 |  |
| Roca-Ginés J, Torres-Navarro I, *et al.* |  | 6 cases had family members with lesions |
| L. Rizzoli, L. Collini, *et al.* |  | 2 cases were sisters |
| El Hachem M, Diociaiuti A, *et al.* |  | 2 cases were brothers |
| Kluckow E, Krieser DM, *et al.* | Chilblains or related health conditions: 0 |  |
| Fabbrocini G, Vastarella M, *et al.* | Autoimmunity: 0 | 3 pairs of siblings |

‡ Coagulation defects in cases and family reported together as 4
